# Adhesion Coefficient Identification of Wheeled Mobile Robot under Unstructured Pavement

**DOI:** 10.3390/s24041316

**Published:** 2024-02-18

**Authors:** Hongchao Zhang, Bao Song, Junming Xu, Hu Li, Shuhui Li

**Affiliations:** 1 School of Mechanical Science & Engineering, Huazhong University of Science and Technology, Wuhan 430074, China; d202187005@hust.edu.cn (H.Z.); songbao@hust.edu.cn (B.S.); xjm0627@163.com (J.X.); 2 North China Vehicle Research Institute, Beijing 100072, China; 3 Guangdong Intelligent Robotics Institute, Dongguan 523830, China; drhuli@foxmail.com; 4 Hubei Key Laboratory of Intelligent Robot, Wuhan Institute of Technology, Wuhan 430205, China

**Keywords:** unstructured pavement, state estimation, extended Kalman filter, equivalent suspension model, pavement adhesion coefficient

## Abstract

Because of its uneven and large slope, unstructured pavement presents a great challenge to obtaining the adhesion coefficient of pavement. An estimation method of the peak adhesion coefficient of unstructured pavement on the basis of the extended Kalman filter is proposed in this paper. The identification accuracy of road adhesion coefficients under unstructured pavement is improved by introducing the equivalent suspension model to optimize the calculation of vertical wheel load and modifying vehicle acceleration combined with vehicle posture data. Finally, the multi-condition simulation experiments with Carsim are conducted, the estimation accuracy of the adhesion coefficient is at least improved by 3.6%, and then the precision and effectiveness of the designed algorithm in the article are verified.

## 1. Introduction

In the field of intelligent vehicles, the unstructured pavement is characterized by complexity and randomness, which more easily causes safety problems and has higher performance requirements for vehicle safety control [1,2,3]. Safety control of vehicles often needs to adjust the force between road surfaces and tires, and the interaction force is also a key and vital factor that affects the stability of the vehicle chassis [4,5]. Therefore, the adhesion coefficient of road surfaces is a vital parameter to obtain accurate motion control of vehicles [6,7], and it also provides a significant input for the decision and planning of intelligent vehicles. It is vital and essential to precisely identify the adhesion coefficients of the unstructured pavements for the safe driving of vehicles.

Recently, a variety of methods have been proposed by domestic and foreign scholars to estimate the road adhesion coefficient. At present, road adhesion coefficient identification mainly consists of cause-based methods as well as effect-based methods [8,9,10,11]. With the development of vehicle intelligence, some special devices (optical sensor or ultrasonic sensor, etc.) are required to measure the factors associated with tires or roads (for example, the deformation and noise of tires, the road texture, etc.), and then identify the road adhesion coefficient in the cause-based methods [12,13]. For example, given the critical and difficult problems about the estimation of adhesion coefficients, Bo Leng et al. proposed fusing vehicle dynamics with machine vision during the estimation [14]. A local binarization algorithm was designed by Du et al. to extract the spatial and texture features of roads gathered by the high-definition cameras [15]. Then, the feature information was introduced into the modified VGGNet to classify the road adhesion level. Herrmann T. et al. adopted an on-board camera and lidar to first estimate the road adhesion coefficient and then collect it based on dynamic information [16]. In a word, the estimation accuracy of cause-based methods is high, but the special measuring equipment in the experiment is expensive and susceptible to fog, rain, and snow.

Effect-based estimation methods use sensors to study tire and road-related factors (for example, the deformation and noise of tires, the road texture, etc.). Then the road adhesion coefficient is calculated by these factors. The estimation methods based on the curve of the adhesion coefficient and the slip rate [17], together with the relationship between the right torque and the side deflection angle of tires [18], are common in the vehicle field, which only needs the common on-board sensors, such as wheel speed sensors and attitude sensors. For example, aligning the torque and the side-slip angle or the relationship description between the tire lateral force is performed through linearization [19], Brush [20,21], TMsimple [22], Pacejka [23], and Burckhardt [24] to identify adhesion coefficients under the condition of lateral movements of the vehicle. However, such a method based on the response recognition of tires is affected by many external uncertainties due to the complexity of the generation mechanism of the tire noise, and sometimes it is insurmountable to accurately identify the adhesion coefficients. Recently, some identification methods on the basis of visual information have been proposed in the current study [25,26,27,28]. For example, given the nondeterminacy of kinematic models and deep-learning models, an image-based fusion estimation method by virtue of the virtual sensing theory was put forward to exactly realize the identification of the road surface condition in reference [25]. However, these visual information-based methods are susceptible to light. In addition, scholars make the best use of different vehicle dynamics models [29,30,31,32,33,34] and various kinds of filters [35,36,37,38] to estimate the adhesion coefficient. For example, the vehicle dynamic model, the tire model, and the wheel model were introduced into the unscented Kalman filter to accurately identify the adhesion coefficient of roads in reference [36]. According to the similarity principle and the adaptive square root cubature Kalman filter, a longitudinal-lateral cooperative estimation algorithm was designed to identify the state of vehicles and the adhesion coefficients of four-wheel independent drive electric vehicles in reference [38]. In short, most of the above algorithms build the vehicle dynamics model with the seven-degree-of-freedom (VDMSDF) and simulate it with the Carsim 2019.0. The complexity and freedom of vehicle models provided by Carsim are much higher than those of the commonly used VDMSDF. Real data should be directly used to replace some parameters that are difficult to fit with the models, which are closer to the actual vehicle model. In addition, the filters, by virtue of two types of models, are adopted to accurately estimate the adhesion coefficients of the pavement under the coupled conditions of lateral and longitudinal forces, which is simple to solve and has fast convergence speed. However, this type of filter-based method does not fully consider the surface roughness and the large slope characteristics of the unstructured pavement, and still faces some challenges:

1. The existing algorithm regards the body and the wheel as a rigid connection, which leads to a large error in the calculated vertical load of the tire under the conditions of the uneven road.
2. The existing algorithms often ignore the influence of gravity on the body acceleration, and straightforwardly input the body acceleration into the observer as an observation quantity, which makes the algorithm unable to accurately estimate the road surface with a certain slope.

Therefore, considering the effects of vertical static and dynamic loads on vehicle acceleration, an estimation method of adhesion coefficients of the pavement on the basis of the extended Kalman filter is proposed for the above issue. The paper primarily investigates the identification of adhesion coefficients under uneven and slope pavement, and it is assumed that all tires together with all wheels are the same. So, the main contributions and highlights of the proposed method are highlighted in the following sentences:

3. The impact of vehicle acceleration on the vertical load is fully considered in the vertical load model of vehicles based on the equivalent suspension model, which can depict road conditions by rule and line.
4. The dynamics model of vehicles established in this paper no longer regards the body and the wheel as rigid body connections, which can more accurately depict the dynamic characteristics of vehicles.
5. By virtue of the extended Kalman filter and the improved dynamic model, the estimation algorithm of adhesion coefficient identification on the unstructured pavement designed in this paper can not only adapt to the non-structural and high-slope pavement but also improve the identification accuracy of adhesion coefficients.

The rest of the paper is approximately structured as follows: In Section 2, the improved dynamics model is discussed first, which is foundation of the system dynamics model in the later content. In Section 3, the identification of adhesion coefficients on the basis of the extended Kalman filter is designed to estimate the tire-road peak adhesion coefficient. In Section 4, the designed identification algorithm in this paper is proven through simulation tests. Finally, the conclusion as well as the application scenario of the proposed method is given.

## 2. Improved Dynamics Model

The adhesion coefficient is related to the force of the tire. Therefore, this paper gradually analyzes the forces from the whole vehicle to the tires and then decomposes the forces from tires to the vertical load. The relationship between the adhesion coefficient and the force of the tire is established through the force analysis in the above steps. Therefore, this chapter will expand the description from the dynamics model of vehicles, tire models and vertical load in turn.

### 2.1. Improved Vehicle Dynamics Model

The vehicle dynamics model can simulate and analyze the motion states of vehicles under different driving scenarios and represent the mathematical relationship among different control inputs, environmental inputs, and vehicle responses during driving, which is the basis of the research for adhesion coefficient identification. At present, the VDMSDF is commonly applied to research on adhesion coefficient identification. It combines the vehicle dynamics model with three degrees of freedom and the wheel model. In addition, the rotational degree of freedom for four wheels is considered, except for the freedom of longitudinal, transverse, and yaw.

**Figure 1:**
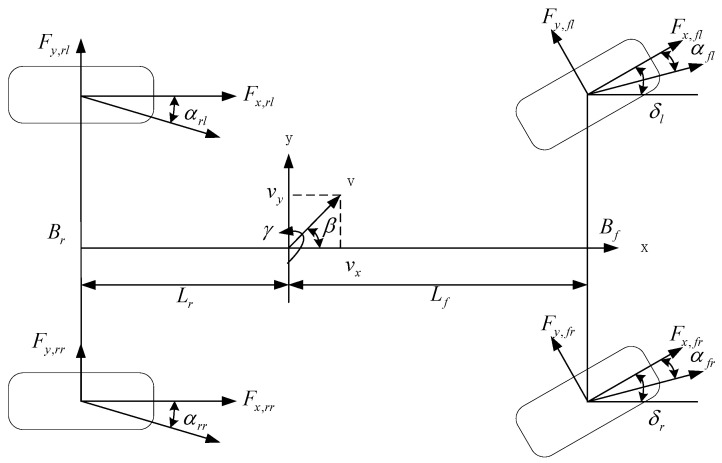
Vehicle dynamics model.

Given Figure 1, the following equation can be calculated according to the vehicle dynamics model:

(1) $${\dot{v}}_{x}-v_{y}\omega_{z}=a_{x}$$

(2) $${\dot{v}}_{y}+v_{x}\omega_{z}=a_{y}$$

The longitudinal dynamic formula is approximately described as the following equation:

(3) $$m\left({\dot{v}}_{x}-v_{y}\omega_{z}\right)=F_{x,fl}\cos\delta +F_{x,fr}\cos\delta +F_{x,rl}+F_{x,rr}-F_{y,fl}\sin\delta -F_{y,fr}\sin\delta$$

Similarly, the lateral dynamics equation can be obtained as follows:

(4) $$m\left({\dot{v}}_{y}+v_{x}\omega_{z}\right)=F_{x,fl}\sin\delta +F_{x,fr}\sin\delta +F_{y,rl}+F_{y,rr}+F_{y,fl}\cos\delta +F_{y,fr}\cos\delta$$

The yaw motion dynamics equation can be calculated in the same way:

(5) $$\begin{array}{l} I_{z}{\dot{\omega }}_{z}=\frac{B_{f}}{2}\left(F_{x,fr}\cos\delta -F_{x,fl}\cos\delta -F_{y,fr}\sin\delta +F_{y,fl}\sin\delta \right)+\frac{B_{r}}{2}\left(F_{x,rr}-F_{x,rl}\right)\\ \;\;+L_{f}\left(F_{x,fl}\sin\delta +F_{y,fl}\cos\delta +F_{y,fr}\sin\delta +F_{y,fl}\sin\delta \right)-L_{r}\left(F_{y,rl}+F_{y,rr}\right) \end{array}$$

where $v_{x}$ and $v_{y}$ are the longitudinal speed and the lateral speed, respectively. $\omega_{z}$ is the angular velocity of the yaw. δ is the angle of the front wheel. m is the total vehicle mass, $F_{x,ij}(ij=fl,fr,rl,rr)$ is the longitudinal force of the tire on the wheels. $F_{y,ij}(ij=fl,fr,rl,rr)$ is the transverse force for four-wheel tires. The moment of inertia is set to $I_{z}$ when vehicles rotate about the z axis. The front and rear tracks are assumed as $B_{f}$ and $B_{r}$, respectively. The distances from the center of mass to the front and rear axles are respectively set to $L_{f}$ and $L_{r}$.

The above model is only suitable for the horizontal road. When the vehicle is driving on a slope road, gravity affects the acceleration of the vehicle. In this case, there is also acceleration caused by gravity, except for the acceleration caused by tire stress. Therefore, it is indispensable to take the influence of gravity into consideration and modify the above dynamics expressions.

It is vital and necessary to take the effects of gravity into account on a slop road. The acceleration component caused by the tire force is calculated by combining the attitude and acceleration of the vehicles.

(6) $$\left\{\begin{array}{c} a_{Fx}=a_{x}-g\sin\theta \\ a_{Fy}=a_{y}-g\sin\phi \end{array}\right.$$

The longitudinal dynamics equation and the transverse dynamics equation are calculated as follows:

(7) $$ma_{Fx}=F_{x,fl}\cos\delta +F_{x,fr}\cos\delta +F_{x,rl}+F_{x,rr}-F_{y,fl}\sin\delta -F_{y,fr}\sin\delta$$

(8) $$ma_{Fy}=F_{x,fl}\sin\delta +F_{x,fr}\sin\delta +F_{y,rl}+F_{y,rr}+F_{y,fl}\cos\delta +F_{y,fr}\cos\delta$$

where $a_{Fx}$ is the longitudinal acceleration component caused by the tire stress, $a_{Fy}$ is the transverse acceleration component caused by the tire stress, ϕ is the roll angle of vehicles, θ is the pitch angle of vehicles.

Given the established dynamics model for vehicles, it is necessary to further calculate the longitudinal and lateral forces of tires by the relationship of the wheel force.

### 2.2. Dugoff Tire Model

The tire model describes the relationship between the tire force and the motion parameters of wheels through mathematical relations. In other words, it is the relationship between the inputs of tires and the outputs of tires under different road conditions. The longitudinal force, the transverse force, and the righting moment in the tire model are usually calculated by the input parameters such as the slip rate, the side deflection angle, and the vertical load.

The Dugoff tire model can describe the relationship between the road adhesion coefficient and the lateral and longitudinal forces. Meanwhile, this model can directly calculate the longitudinal force and the lateral force according to the slip rate and the side deflection angle of the vehicle. The Dugoff tire model is expressed as follows [39,40,41,42]:

(9) $$F_{x}=C_{x}\frac{\lambda }{1+\lambda }\times f\left(L\right)$$

(10) $$F_{y}=C_{y}\frac{\tan\alpha }{1+\lambda }\times f\left(L\right)$$

where L and $f\left(L\right)$ can be respectively expressed as follows:

(11) $$L=\frac{\mu F_{z}\left(1+\lambda \right)}{2\sqrt{{C_{x}}^{2}\lambda^{2}+{C_{y}}^{2}\tan^{2}\alpha }}$$

(12) $$f\left(L\right)=\left\{\begin{array}{c} L\left(2-L\right)\\ 1 \end{array}\right.\begin{array}{c} ,\\ , \end{array}\begin{array}{c} L<1\\ L\geq 1 \end{array}$$

$C_{x}$ and $C_{y}$ are the longitudinal and lateral stiffness of tires, respectively. λ is the slip rate. α is the lateral drift angle of tires. L is the nonlinear characteristic parameter used to express the slip of tires.

In order to effectively identify the adhesion coefficients, it is necessary and vital to reorganize the Dugoff tire model so that the dominant relational expression between the road adhesion coefficient and the Dugoff tire model can be directly obtained. Let $C_{x}^{0}=C_{x}/\mu F_{z}$ and $C_{y}^{0}=C_{y}/\mu F_{z}$. Considering the calculation formulas for the lateral and longitudinal forces in the Dugoff tire model, a nonlinear characteristic parameter of tires under slipping L can be expressed:

(13) $$L=\frac{1+\lambda }{2\sqrt{{\left(C_{x}^{0}\right)}^{2}\lambda^{2}+{\left(C_{y}^{0}\right)}^{2}\tan^{2}\alpha }}$$

Next, the lateral force and longitudinal force of tires can be calculated by the following equations:

(14) $$F_{x}=\mu F_{z}C_{x}^{0}\frac{\lambda }{1+\lambda }\times f\left(L\right)$$

(15) $$F_{y}=\mu F_{z}\cdot C_{y}^{0}\frac{\tan\alpha }{1+\lambda }\times f\left(L\right)$$

It can be seen from the calculation formula of L that this deformation does not change the value of L, nor does it change the calculation of lateral and longitudinal forces. Moreover, the product of parameters in the formula except for adhesion coefficients after deformation is set to the normalized force, namely $F_{x}^{0}$ and $F_{y}^{0}$. Then the following formula can be obtained:

(16) $$F_{x}=\mu {F_{x}}^{0}$$

(17) $$F_{y}=\mu {F_{y}}^{0}$$

Therefore, the actual tire force can be expressed as the dynamic equation through the normalized force and the adhesion coefficient of roads, given the corresponding relationship between the force of tires and the adhesion coefficient in the Dugoff tire model.

It is obvious that the model in Equations (17) and (18) can be applied to the algorithm after the mathematical deformation, which is conducive to the subsequent research on adhesion coefficient identification. Therefore, the deformable Dugoff tire model is adopted in identification algorithms.

Therefore, considering the side deflection angles, the slip rate, and the vertical load, the Dugoff normalized force can be calculated as follows:

(18) $$\left\{\begin{array}{c} {F_{x}}^{0}=F_{z}\cdot C_{x}\frac{\lambda }{1+\lambda }\times f\left(L\right)\\ {F_{y}}^{0}=F_{z}\cdot C_{y}\frac{\tan\alpha }{1+\lambda }\times f\left(L\right) \end{array}\right.$$

Since the force of tires changes with the speed of vehicles during the driving process, add a speed correction $1-\varepsilon v_{x}\sqrt{{C_{x}}^{2}\lambda^{2}+{C_{y}}^{2}\tan^{2}\alpha }$ to the nonlinear characteristic parameters of tires under slipping L, namely:

(19) $$L=\frac{\mu F_{z}\left(1+\lambda \right)}{2\sqrt{{C_{x}}^{2}\lambda^{2}+{C_{y}}^{2}\tan^{2}\alpha }}\left(1-\varepsilon v_{x}\sqrt{{C_{x}}^{2}\lambda^{2}+{C_{y}}^{2}\tan^{2}\alpha }\right)$$

where ε is the influence factor of the velocity. It is only related to the material and structure of tires and can be utilized to describe the effect of the slip speed on the force of tires. In addition, the Dugoff tire model requires that the vertical load, the lateral drift angle of tires, and the slip rate be known, so that the slip rate and the lateral drift angle of tires can be calculated from the state parameters of vehicles.

Firstly, the vehicle speed signal, the front wheel angle signal, and the yaw angle speed signal are obtained by the sensor installed on the vehicles. On this basis, the lateral drift angle of wheels $\alpha_{ij}$ can be calculated using the following equations:

(20) $$\alpha_{fl}=-\left(\delta -\arctan\frac{v_{y}+L_{f}\omega_{z}}{v_{x}-\frac{B_{f}\omega_{z}}{2}}\right)$$

(21) $$\alpha_{fr}=-\left(\delta -\arctan\frac{v_{y}+L_{f}\omega_{z}}{v_{x}+\frac{B_{f}\omega_{z}}{2}}\right)$$

(22) $$\alpha_{rl}=\arctan\frac{v_{y}-L_{r}\omega_{z}}{v_{x}-\frac{B_{r}\omega_{z}}{2}}$$

(23) $$\alpha_{rr}=\arctan\frac{v_{y}-L_{r}\omega_{z}}{v_{x}+\frac{B_{r}\omega_{z}}{2}}$$

In Equations (20)–(23), $v_{x}$ and $v_{y}$ are the longitudinal speed and the transverse speed of vehicles, respectively. $\omega_{z}$ is the yaw angle speed of vehicles. δ is the angle of the front wheel. $B_{f}$ and $B_{r}$ are the front wheel base and the rear wheel base of vehicles, respectively. $L_{f}$ and $L_{r}$ are the distances from the center of mass to the front axles and the rear axles, respectively. The speed in the longitudinal axis direction of the core wheel under the wheel coordinate system $v_{wx,ij}$ is calculated as follows:

(24) $$v_{wx,fl}=\left(v_{x}-\frac{B_{f}\omega_{z}}{2}\right)\cos\delta +\left(v_{y}+L_{f}\omega_{z}\right)\sin\delta$$

(25) $$v_{wx,fr}=\left(v_{x}+\frac{B_{f}\omega_{z}}{2}\right)\cos\delta +\left(v_{y}+L_{f}\omega_{z}\right)\sin\delta$$

(26) $$v_{wx,rl}=v_{x}-\frac{B_{r}\omega_{z}}{2}$$

(27) $$v_{wx,rr}=v_{x}+\frac{B_{r}\omega_{z}}{2}$$

The wheel speed is obtained through the wheel speed sensor. The slip rate of wheels $\lambda_{ij}$ is obtained as the following equation:

(28) $$\lambda_{ij}=\frac{\omega_{w,ij}R_{W}-v_{wx,ij}}{\max\left(\omega_{w,ij}R_{W},v_{wx,ij}\right)}$$

where $\omega_{w,ij}$ represents the speed of wheels, $R_{W}$ is the radius of wheels, ij∈{fl,fr,rl,rr}. In addition to the acquisition of the lateral drift angle and the slip rate, it is also necessary to analyze the vertical load during the movement. The calculation of the vertical load can be discussed in the following chapter.

### 2.3. Improved Vertical Load Model

It is necessary for the identification of the adhesion coefficients to obtain the side deflection angle of tires, the vertical load, together with the slip rate. Under a non-structural road, the vertical load is often affected by the vehicle’s vertical acceleration, so it is necessary to build an estimation model of the vertical load of tires with higher accuracy. A vertical load model based on the equivalent suspension is established in this paper.

Firstly, the vertical static load of wheels $F_{w\_ij}$ can be calculated by the self-propelled parameters:

(29) $$F_{w\_fl}=\frac{1}{2}\times \frac{m_{b}gL_{r}}{L_{f}+L_{r}}+m_{w}g$$

(30) $$F_{w\_rl}=\frac{1}{2}\times \frac{m_{b}gL_{r}}{L_{f}+L_{r}}+m_{w}g$$

(31) $$F_{w\_rl}=\frac{1}{2}\times \frac{m_{b}gL_{f}}{L_{f}+L_{r}}+m_{w}g$$

(32) $$F_{w\_rr}=\frac{1}{2}\times \frac{m_{b}gL_{f}}{L_{f}+L_{r}}+m_{w}g$$

In Equations (29)–(32), $m_{b}$ is the spring mass of the vehicle. $m_{W}$ is the wheel mass; The acceleration of the gravity is set to g.

**Figure 2:**
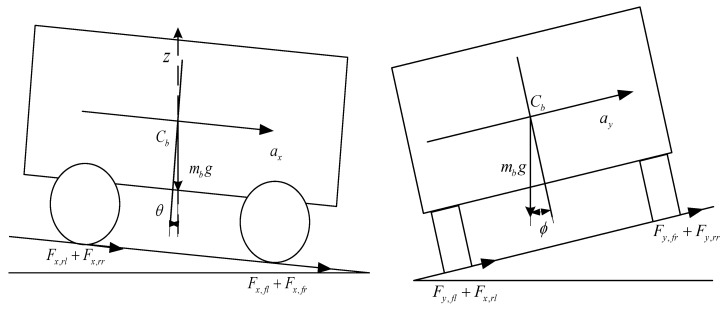
Roll and pitch motion models of vehicles.

In Figure 2, based on the models of the roll motion and the pitch motion of vehicles, the displacement, together with the speed of suspensions, is analyzed as follows:

The displacements of the suspension for each wheel are calculated by the roll and pitch angle of the body together with the centroid displacement:

(33) $$z_{s\_fl}=z_{b}+\frac{B_{f}}{2}\sin\phi -L_{f}\sin\theta$$

(34) $$z_{s\_fr}=z_{b}-\frac{B_{f}}{2}\sin\phi -L_{f}\sin\theta$$

(35) $$z_{s\_rl}=z_{b}+\frac{B_{r}}{2}\sin\phi +L_{r}\sin\theta$$

(36) $$z_{s\_rr}=z_{b}-\frac{B_{r}}{2}\sin\phi +L_{r}\sin\theta$$

In Equations (33)–(36), $z_{b}$ is the vertical displacement of the vehicle centroid. The roll angle of the body is set as ϕ. Set θ as the pitch angle of the body. The suspension velocity is obtained by differentiating the suspension displacement:

(37) $${\dot{z}}_{s\_fl}={\dot{z}}_{b}+\frac{B_{f}}{2}\cos\phi \times \dot{\phi }-L_{f}\cos\theta \times \dot{\theta }$$

(38) $${\dot{z}}_{s\_fr}={\dot{z}}_{b}-\frac{B_{f}}{2}\cos\phi \times \dot{\phi }-L_{f}\cos\theta \times \dot{\theta }$$

(39) $${\dot{z}}_{s\_rl}={\dot{z}}_{b}+\frac{B_{r}}{2}\cos\phi \times \dot{\phi }+L_{r}\cos\theta \times \dot{\theta }$$

(40) $${\dot{z}}_{s\_rr}={\dot{z}}_{b}-\frac{B_{r}}{2}\cos\phi \times \dot{\phi }+L_{r}\cos\theta \times \dot{\theta }$$

The vertical acceleration and the vertical displacement of the wheel are obtained by the accelerometer and the angle sensor, respectively, and then the vertical speed signals are processed. The dynamic suspension force is calculated as follows:

(41) $$F_{dzs\_fl}=k_{f}\left(z_{w\_fl}-z_{s\_fl}\right)+c_{f}\left({\dot{z}}_{w\_fl}-{\dot{z}}_{s\_fl}\right)$$

(42) $$F_{dzs\_fr}=k_{f}\left(z_{w\_fr}-z_{s\_fr}\right)+c_{f}\left({\dot{z}}_{w\_fr}-{\dot{z}}_{s\_fr}\right)$$

(43) $$F_{dzs\_rl}=k_{r}\left(z_{w\_rl}-z_{s\_rl}\right)+c_{r}\left({\dot{z}}_{w\_rl}-{\dot{z}}_{s\_rl}\right)$$

(44) $$F_{dzs\_rr}=k_{r}\left(z_{w\_rr}-z_{s\_rr}\right)+c_{r}\left({\dot{z}}_{w\_rr}-{\dot{z}}_{s\_rr}\right)$$

**Figure 3:**
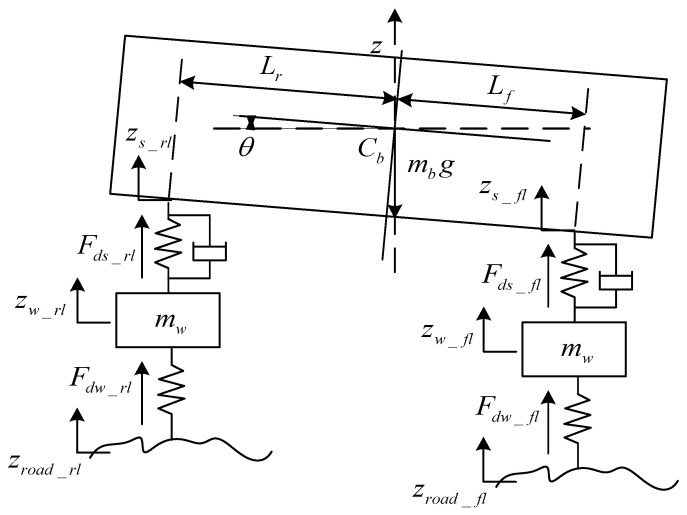
Force analysis of wheels.

In Equations (41)–(44), $z_{w\_ij}$ denotes the vertical displacement of each wheel; $k_{f}$ and $k_{r}$ are the equivalent stiffness coefficients of the suspension, respectively. $c_{f}$ and $c_{r}$ are the equivalent damping coefficients of the suspension, respectively. The force analysis of wheels is shown in Figure 3:

The dynamic vertical load of tires can be calculated by the force of dynamic suspension and wheel acceleration:

(45) $$F_{dw\_fl}=m_{w}{\ddot{z}}_{w\_fl}-F_{ds\_fl}$$

(46) $$F_{dw\_fr}=m_{w}{\ddot{z}}_{w\_fr}-F_{ds\_fr}$$

(47) $$F_{dw\_rl}=m_{w}{\ddot{z}}_{w\_rl}-F_{ds\_rl}$$

(48) $$F_{dw\_rr}=m_{w}{\ddot{z}}_{w\_rr}-F_{ds\_rr}$$

The current vertical load of wheels is obtained by adding the calculated vertical static load and the dynamic load.

## 3. Adhesion Coefficient Identification by Virtue of Extended Kalman Filter

### 3.1. Description of System Equations Based on the Improved Dynamics Model

Considering that the influencing factors of the adhesion are complex and the reproducibility of the adhesion is poor in different scenarios, based on the principle of grasping the main contradiction, the modeling errors caused by the sensor noise, limited/limited sampling time, unmodeled dynamics, and other factors in the paper are equivalent to Gaussian noise in the dynamic model about the road adhesion coefficient. In addition, due to the nonlinearity of the established dynamic equation about the adhesion coefficient, it is necessary to select a suitable filter for estimation. The effectiveness of the extended Kalman filter has been recognized by many scholars and engineers because it can take into account the nonlinear modeling error of dynamic models about the adhesion coefficient. Meanwhile, considering the real-time performance of the extended Kalman filter, this paper selects the extended Kalman filter to identify the adhesion coefficient. Thus, the state equation for the designed vehicle dynamics is given as follows:

(49) $$x\left(t\right)=f\left(x\left(t\right),u\left(t\right),w\left(t\right)\right)=\left[\begin{array}{cccc} 1 & 0 & 0 & 0\\ 0 & 1 & 0 & 0\\ 0 & 0 & 1 & 0\\ 0 & 0 & 0 & 1 \end{array}\right]\times \left[\begin{array}{c} \mu_{fl}\\ \mu_{fr}\\ \mu_{rl}\\ \mu_{rr} \end{array}\right]+w\left(t\right)$$

where w(t) is the process noise, the mean square error is Q, $\mu_{ij}$ is the adhesion coefficient of the four-lane highway.

The actual tire force in the three dynamics equations of vehicles can be written in the form of the normalized force. Then the nonlinear equation is linearized. The measurement equation is expressed as follows:

(50) $$\begin{array}{l} y\left(t\right)=h\left(x\left(t\right),v\left(t\right)\right)\\ =\left[\begin{array}{cccc} h(1,1) & h(1,2) & \frac{{F^{0}}_{xrl}}{m} & \frac{{F^{0}}_{xrr}}{m}\\ h(2,1) & h(2,2) & \frac{{F^{0}}_{yrl}}{m} & \frac{{F^{0}}_{yrr}}{m}\\ h\left(3,1\right) & h\left(3,2\right) & h\left(3,3\right) & h\left(3,4\right) \end{array}\right]\times \left[\begin{array}{c} \mu_{fl}\\ \mu_{fr}\\ \mu_{rl}\\ \mu_{rr} \end{array}\right]+v\left(t\right) \end{array}$$

where

$$h(1,1)=\frac{{F^{0}}_{xfl}\times \cos\delta_{l}-{F^{0}}_{yfl}\times \sin\delta_{l}}{m}$$

$$h(1,2)=\frac{{F^{0}}_{xfr}\times \cos\delta_{r}-{F^{0}}_{yfr}\times \sin\delta_{r}}{m}$$

$$h(2,1)=\frac{{F^{0}}_{xfl}\times \sin\delta_{l}+{F^{0}}_{yfl}\times \cos\delta_{l}}{m}$$

$$h(2,2)=\frac{{F^{0}}_{xfr}\times \sin\delta_{r}+{F^{0}}_{yfr}\times \cos\delta_{r}}{m}$$

$$h\left(3,1\right)=\left(l_{a}\left({F^{0}}_{xfl}\times \sin\delta_{l}+{F^{0}}_{yfl}\times \cos\delta_{l}\right)\right.+\frac{T_{f}}{2}\left({F^{0}}_{xfl}\right.\left.\left.\times \cos\delta_{l}-{F^{0}}_{yfl}\times \sin\delta_{l}\right)\right)/I_{z}$$

$$h\left(3,2\right)=\left(l_{a}\left({F^{0}}_{xfr}\times \sin\delta_{r}+{F^{0}}_{yfr}\times \cos\delta_{r}\right)+\right.\frac{T_{f}}{2}\left({F^{0}}_{xfr}\right.\times \left.\left.\cos\delta_{r}-{F^{0}}_{yfr}\times \sin\delta_{r}\right)\right)/I_{z}$$

$$h\left(3,3\right)=\frac{-\frac{T_{r}}{2}\times {F^{0}}_{xrl}-l_{b}\times {F^{0}}_{yrl}}{I_{z}}$$

$$h\left(3,4\right)=\frac{\frac{T_{r}}{2}\times {F^{0}}_{xrr}-l_{b}\times {F^{0}}_{yrr}}{I_{z}}$$

where R is the mean square error, v(t) is assumed as the process noise, $F_{xij}^{0}$ and $F_{yij}^{0}$ are the longitudinal and lateral normalized forces of tires, respectively, $\delta_{l}$ and $\delta_{r}$ are the angles of left front and right front wheels, respectively.

Assume that all tires are identical and their adhesion coefficients are uniform in this paper. In order to improve the calculation speed, the above state and measurement equations are modified as follows:

(51) $$x\left(t\right)=f\left(x\left(t\right),u\left(t\right),w\left(t\right)\right)=1\times \mu +w\left(t\right)$$

(52) $$y\left(t\right)=h\left(x\left(t\right),v\left(t\right)\right)=\left[\begin{array}{c} h_{1}\\ h_{2}\\ h\left(3,1\right)+h\left(3,2\right)+h\left(3,3\right)+h\left(3,4\right) \end{array}\right]\mu +v\left(t\right)$$

(53) $$h_{1}=\frac{{F^{0}}_{xfl}\times \cos\delta_{l}-{F^{0}}_{yfl}\times \sin\delta_{l}}{m}+\frac{{F^{0}}_{xfr}\times \cos\delta_{r}-{F^{0}}_{yfr}\times \sin\delta_{r}}{m}+\frac{{F^{0}}_{xrl}}{m}+\frac{{F^{0}}_{xrr}}{m}$$

(54) $$h_{2}=\frac{{F^{0}}_{xfl}\times \sin\delta_{l}+{F^{0}}_{yfl}\times \cos\delta_{l}}{m}+\frac{{F^{0}}_{xfr}\times \sin\delta_{r}+{F^{0}}_{yfr}\times \cos\delta_{r}}{m}+\frac{{F^{0}}_{yrl}}{m}+\frac{{F^{0}}_{yrr}}{m}$$

where μ is the road adhesion coefficient, and other symbols are defined in the same way as the above formulas.

### 3.2. Extended Kalman Filter

The detailed iterative process of the extended Kalman filter is as follows: Let ${\hat{x}}_{k-1}$ be the state estimation at time k−1. Set $P_{k-1}$ as the covariance at time k−1. Linearize the equation of state by Taylor expansion at ${\hat{x}}_{k-1}$ and obtain [43,44,45,46]:

(55) $$x_{k}=f\left({\hat{x}}_{k-1},u_{k-1},w_{k-1}\right)+F_{k-1}\left(x_{k-1}-{\hat{x}}_{k-1}\right)+w_{k-1}$$

where $F_{k-1}={\left.\frac{\partial f}{\partial x}\right|}_{{\hat{x}}_{k-1},u_{k-1}}$. Let ${\tilde{x}}_{k}=f\left({\hat{x}}_{k-1},u_{k-1},0\right)$. Similarly, linearize the measurement equation and obtain:

(56) $$z_{k}=h\left({\tilde{x}}_{k},v_{k}\right)+H_{k}\left(x_{k}-{\tilde{x}}_{k}\right)+v_{k}$$

where $H_{k}={\left.\frac{\partial h}{\partial x}\right|}_{{\tilde{x}}_{k}}$. The predicted prior state is

(57) $${\hat{x}}_{k}^{-}=E\left[f\left({\hat{x}}_{k-1},u_{k-1},w_{k-1}\right)+F_{k-1}\left(x_{k-1}-{\hat{x}}_{k-1}\right)+w_{k-1}\right]$$

Since $w_{k-1}$ is the process noise satisfying Gaussian distribution with zero mean value and the estimated state is assumed to be the true value, the following equation can be obtained

(58) $${\hat{x}}_{k}^{-}=f\left({\hat{x}}_{k-1},u_{k-1},0\right)$$

The prior covariance of the state estimation is

(59) $$\begin{array}{l} P_{k}^{-}=E\left[\left(x_{k}-{\hat{x}}_{k}^{-}\right){\left(x_{k}-{\hat{x}}_{k}^{-}\right)}^{T}\right]\\ \;\;=E\left\{\left[F_{k-1}\left(x_{k-1}-{\hat{x}}_{k-1}\right)+w_{k-1}\right]{\left[\left(F_{k-1}\left(x_{k-1}-{\hat{x}}_{k-1}\right)+w_{k-1}\right)\right]}^{T}\right\}\\ \;\;=F_{k-1}P_{k-1}{F_{k-1}}^{T}+Q \end{array}$$

Similarly, the predicted values and the covariance matrix for measurements are respectively as following:

(60) $$\left\{\begin{array}{l} {\hat{z}}_{k}=h\left({\tilde{x}}_{k},0\right)\\ P_{zz,k}=H_{k}P_{k}^{-}H_{k}^{T}+R \end{array}\right.$$

The cross-covariance matrix between the state and the measurement is

(61) $$\begin{array}{l} P_{xz,k}=E\left[\left(x_{k}-{\hat{x}}_{k}^{-}\right){\left(z_{k}-{\hat{z}}_{k}\right)}^{T}\right]\\ \;\;=E\left\{\left(x_{k}-{\hat{x}}_{k}^{-}\right){\left[\left(H_{k}\left(x_{k}-{\hat{x}}_{k}^{-}\right)+v_{k}\right)\right]}^{T}\right\}\\ \;\;=P_{k}^{-}H_{k}^{T} \end{array}$$

The gain matrix of the state is

(62) $$K_{k}=\frac{P_{xz,k}}{P_{zz,k}}=\frac{P_{k}^{-}H_{k}^{T}}{H_{k}P_{k}^{-}H_{k}^{T}+R}$$

Then the estimation of the state at time k is

(63) $${\hat{x}}_{k}={\hat{x}}_{k}^{-}+K_{k}\left(z_{k}-{\hat{z}}_{k}\right)$$

The covariance matrix of the state estimation is

(64) $$\begin{array}{l} P_{k}^{-}=E\left[\left(x_{k}-{\hat{x}}_{k}\right){\left(x_{k}-{\hat{x}}_{k}\right)}^{T}\right]\\ \;\;=E\left\{\left[x_{k}-{\hat{x}}_{k}^{-}-K_{k}\left(z_{k}-{\hat{z}}_{k}\right)\right]{\left[x_{k}-{\hat{x}}_{k}^{-}-K_{k}\left(z_{k}-{\hat{z}}_{k}\right)\right]}^{T}\right\}\\ \;\;=\left(I-K_{k}H_{k}\right)P_{k}^{-}{\left(I-K_{k}H_{k}\right)}^{T}+K_{k}RK_{k}^{T}\\ \;\;=\left(I-K_{k}H_{k}\right)P_{k}^{-} \end{array}$$

**Figure 4:**
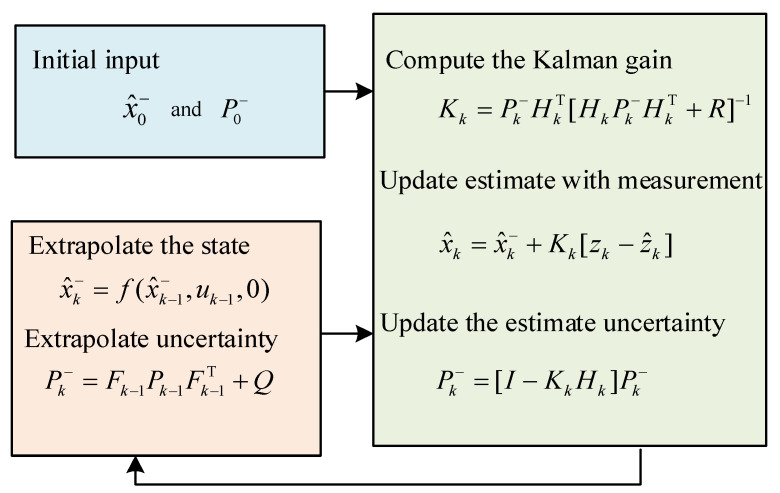
Algorithm flow chart of extended Kalman filter.

The flow chart of the extended Kalman algorithm is shown in Figure 4. It mainly includes two modules, of which one is the update module and the other is the prediction module. The filter integrates the received sensor signals and first updates the time to achieve the prior estimation of state. After that, update the measurements to gain the posterior estimate according to the measurements. The equation modification is done by updating the equations of the state and measurement, which completes one iteration. With continuous iteration over time, the parameters are constantly modified to complete the precise estimation of adhesion coefficients.

### 3.3. Identification Principle of Road Adhesion Coefficients

**Figure 5:**
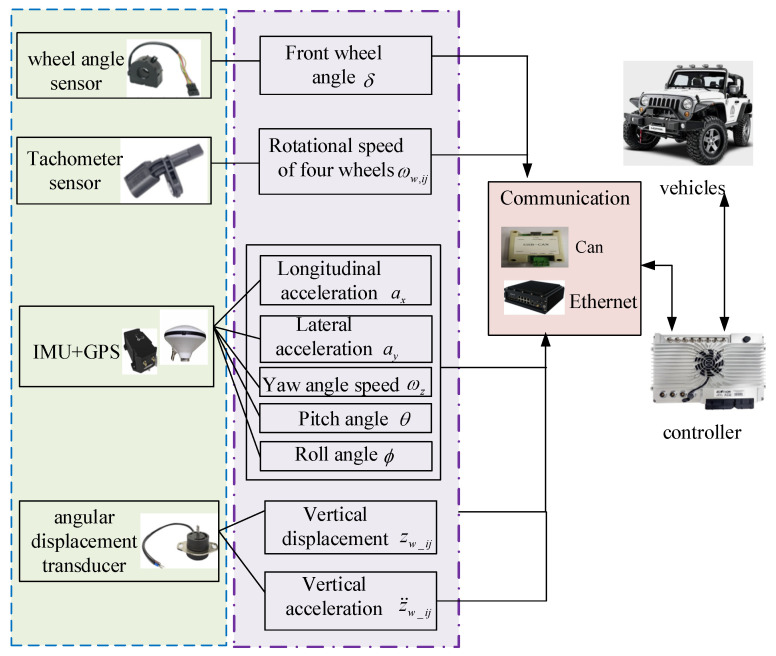
Flow chart of data acquisition.

According to the study about the dynamics model of vehicles, the mathematical expression between the response signal of vehicles and the adhesion coefficients is established. According to Figure 5, the necessary data for the identification of adhesion coefficients are collected by vehicle sensors. For example, the wheel angle sensor is used to capture the angles of four wheels. The tachometer sensor is used to gather the rotational speed of four wheels. Then the collected data is sent through the vehicle communication network. The controller makes full use of the collected data to identify the adhesion coefficient and then conducts the corresponding algorithm to drive the vehicles.

**Figure 6:**
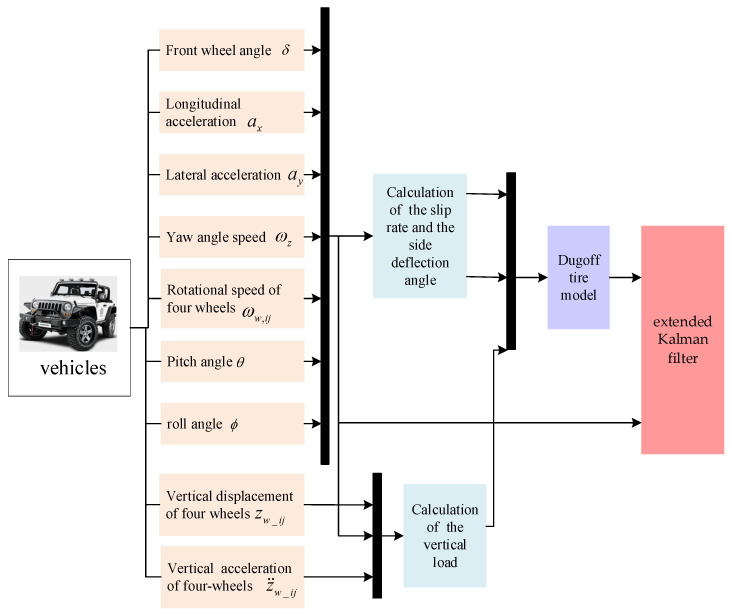
Flow chart of adhesion coefficient identification algorithms.

The overall process of the adhesion coefficient estimation methods is given, just like in Figure 6. The identification algorithm for the road adhesion coefficient is designed by the mathematical relationship in Figure 6. Firstly, the signal gathered by the vehicle sensors is processed. Secondly, the slip rate and the side deflection angle are calculated by the wheel speed, the longitudinal and transverse speed, the front wheel angle, and other parameters. The signals of the wheel vertical displacement, the vertical acceleration, the pitch of vehicles, and the roll angle are transmitted to the vertical load calculation module for processing. Thirdly, the vertical loads, the output slip rate, and the side deflection angles in each parameter calculation module are used as input parameters to calculate the normalized force through the Dugoff tire model. Finally, the normalized force, the vehicle acceleration signal, and the wheel angle are transferred to the extended Kalman filter. The prior state estimation is obtained through the prediction part of the filter. And the measurement is updated by the respective measured values for correction, and the identification result of the adhesion coefficient of roads is updated and corrected through iterations.

## 4. Simulation Results and Analysis

CarSim is a special simulation software in the vehicle field that can simulate the vehicle’s response to driver, road surface, and aerodynamic input. And Carsim is widely utilized in modern automobile control systems. Based on the recognition and wide application of Carsim in the industry, this paper uses Carsim to build a simulation environment to simulate the vehicle response under different road surfaces and scenarios with different control inputs and enters vehicle response signals into the algorithm module built in Matlab/Simulink as input signals. Through the joint simulation, the identification effects of the traditional seven-DOF vehicle dynamics model and the improved algorithm proposed in this paper are compared under different road surfaces and driving scenarios.

### 4.1. Simulation Scenario and Parameter Setting

When designing the algorithm, the applicability of the algorithm under slopes and uneven road surfaces is optimized. In order to ensure its effectiveness, the vehicle model and wheel vertical load model are first verified. Finally, the experiments of pavement adhesion coefficient estimation under different working conditions are carried out. The sensor acquisition principle of this project is shown in Figure 5. Due to the limitations of laboratory equipment, this project is only verified by simulation experiments in CarSim. For the above three scenarios, the simulation parameters are described in the following two sections.

#### 4.1.1. Simulation Parameters in the Vertical Load Model and the Dynamics Model

**Figure 7:**
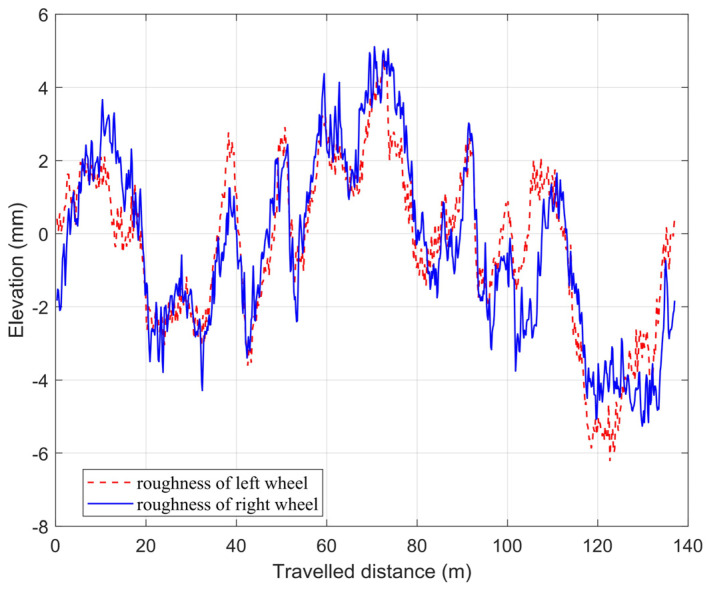
Road roughness.

CarSim can directly output the actual tire force. In the co-simulation environment, the angle of front wheels, the longitudinal force of tires, and the transverse force of tires are input into the dynamic equation of vehicles in the directions of the longitudinal, transverse, and yaw, respectively, to solve their accelerations. In order to verify the applicability of the established vehicle dynamics model and the vertical load model under the slope road, a simulation environment is built in CarSim, and the slope is set at 0.2 m/1 m. Experimental parameters are assumed, just like in Table 1. In addition, the road surface is supposed to have certain unevenness (see Figure 7) and slope to simulate the characteristics of a non-structural road surface.

#### 4.1.2. Simulation Parameters in the Experimental Verification of the Adhesion Coefficient Identification

**Figure 8:**
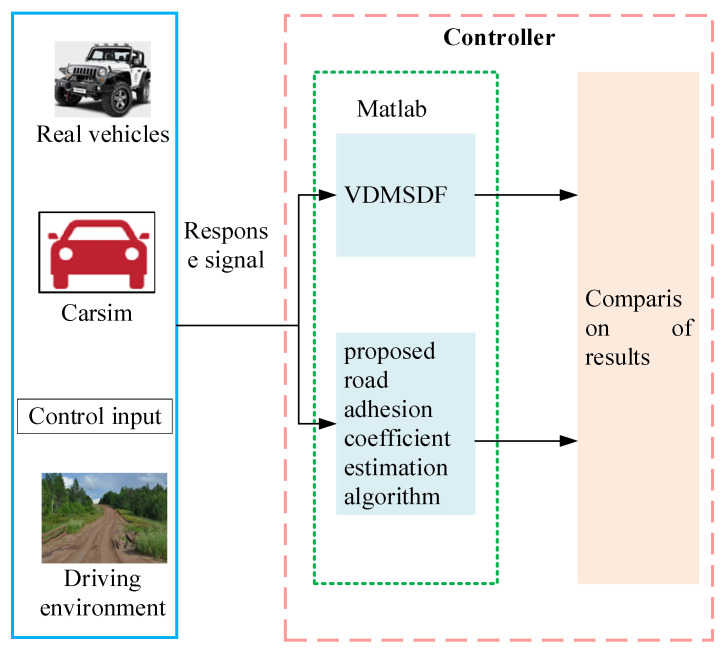
Experimental flow chart of adhesion coefficient identification for pavements.

For the sake of verifying the effectiveness of the developed algorithm, the corresponding models are constructed in the MATLAB/Simulink environment. The vehicle motion parameters are set by CarSim, the actual vehicle driving scenario is simulated, and the required parameters are estimated by the extended Kalman filter. The process of experimentation for the identification of specific pavement adhesion coefficients is presented in Figure 8. The vehicle model, the driving environment, and the control input are set by CarSim to satisfy the simulation requirements of different environments and different working conditions, and then the vehicle response signal is input into MATLAB to identify and compare with our method and traditional methods (VDMSDF). It should be noted that the simulation environment of CarSim can be tested with real cars if conditions permit. In order to verify the effectiveness of the adhesion coefficient identification algorithm, the scenarios with and without steering and braking are analyzed and verified, respectively.

**Case 1: No steering and braking scenario**

According to various working conditions, CarSim is used to set different adhesion coefficients, select the same vehicle model for simulation, and output the corresponding vehicle response signals. The algorithm model is built in the MATLAB/Simulink environment to receive vehicle responses and compare the true road adhesion coefficient with the identification value. The project designs the following three sets of simulation experiments to test the identification results of high, medium, and low adhesion coefficients, respectively.

The method proposed in this paper is primarily applied to the autonomous driving of vehicles in complex off-road, high-speed scenarios. Therefore, the initial speeds of three simulation experiments are respectively set at 100 km/h to meet the requirements of high-speed driving, and the road surface is set to have certain unevenness (see Figure 7) and slope to simulate the characteristics of non-structural road surfaces, and the specific parameter is displayed in Table 2.

**Case 2: Steering and braking scenario**

For the sake of testing the estimation effect of the method in different scenarios, a vehicle steering braking scenario is built in CarSim for experiments. Also, three sets of simulation experiments in Table 3 are designed to test the estimation results of different adhesion coefficients in the turning scene, respectively. The road surface is set to have certain unevenness (see Figure 7). So the slope is set at 0.2 m/1 m to aim at proving the validity of the algorithm under different slope conditions.

**Case 3: Variable adhesion coefficient scenario**

The road surface drive on by vehicles usually covers a variety of types. Due to different ground materials and other factors, the adhesion force received by the vehicle during the driving process can also change accordingly. Therefore, in addition to the two above scenes, this paper also considers a scene with a varying adhesion coefficient to verify the effectiveness of the algorithm. The true adhesion coefficient is assumed to be 0.3 for 0–1.7 s and 0.6 for 1.7 s to 4 s. Other parameter settings are the same as those in Case 1 and Case 2.

### 4.2. Experimental Results and Analysis about the Dynamics Model of Vehicles

**Figure 9:**
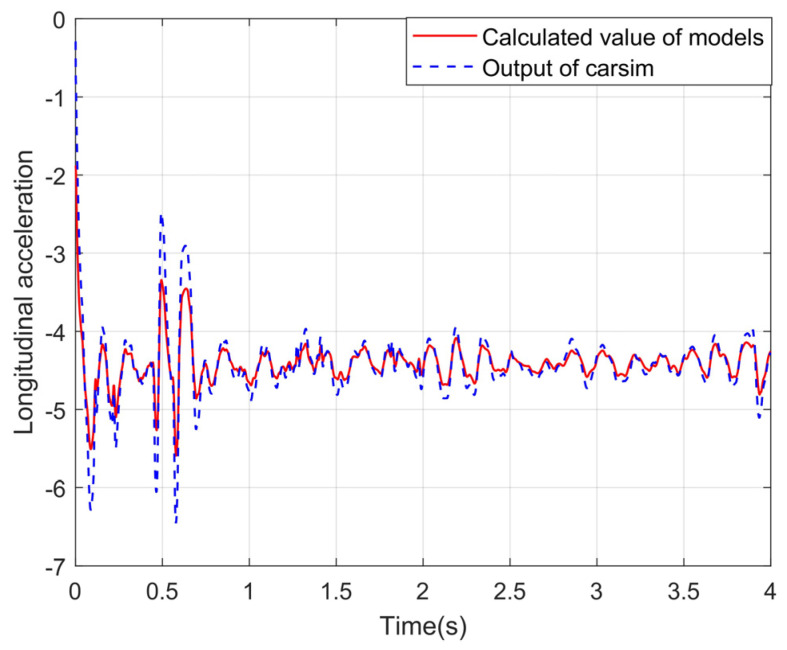
Longitudinal acceleration under the slope road.

**Figure 10:**
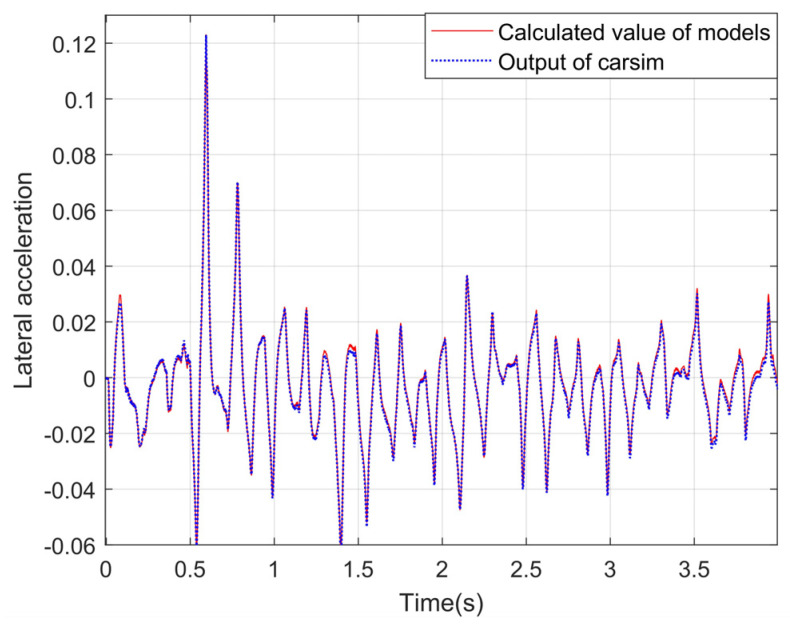
Lateral acceleration under the slope road.

**Figure 11:**
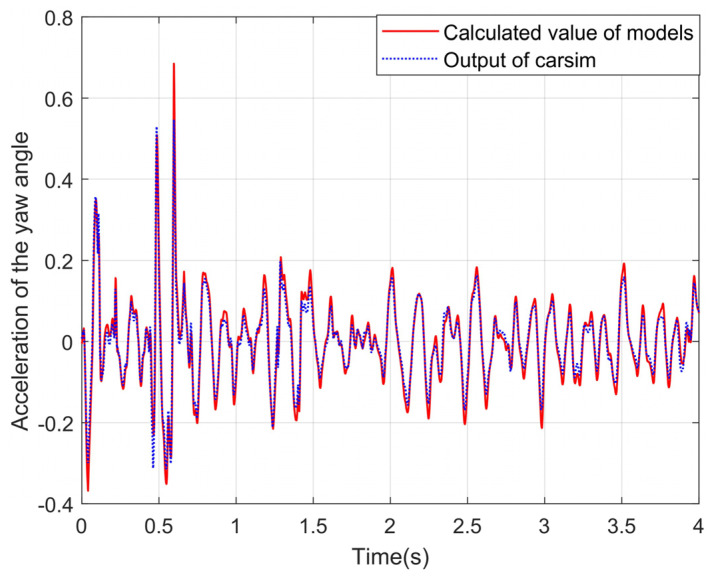
Acceleration of yaw angles under the slope roads.

The longitudinal, transverse, and yaw accelerations of CarSim are outputs for comparison. The correctness of the proposed vehicle dynamics model can be tested by comparison. The experimental curves are shown in Figure 9, Figure 10 and Figure 11.

It can be seen from the above experimental curves that the calculated values of the lateral and longitudinal acceleration, together with the acceleration of the yaw angle, can maintain a good consistency with the real values in the changing trend, which indicates that the established vehicle model can better reflect the motion status of vehicles under the simulation condition of the slope road. Since the equivalent suspension model is introduced in this paper to optimize the calculation of vertical wheel load and to correct vehicle acceleration combined with vehicle pose data, the proposed algorithm in this paper improves the accuracy of the vehicle dynamics model under uneven and sloping road surfaces.

### 4.3. Experimental Results and Analysis about the Vertical Load Model

**Figure 12:**
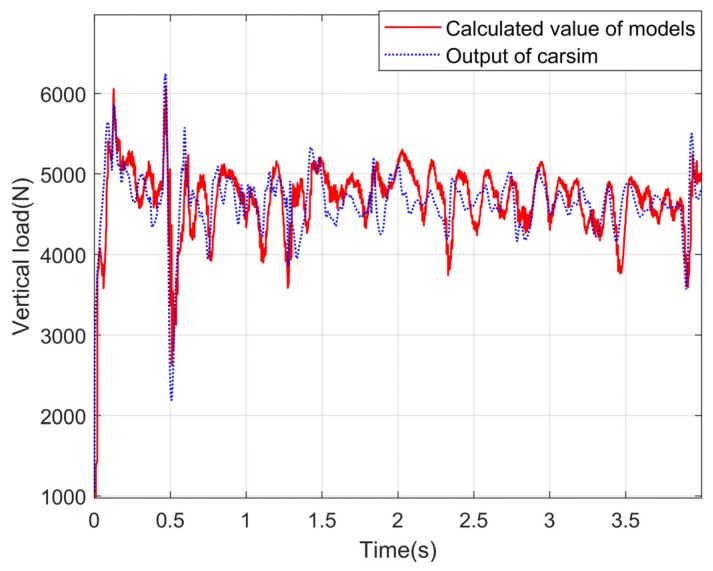
Vertical loads for left front wheels under the slope road.

**Figure 13:**
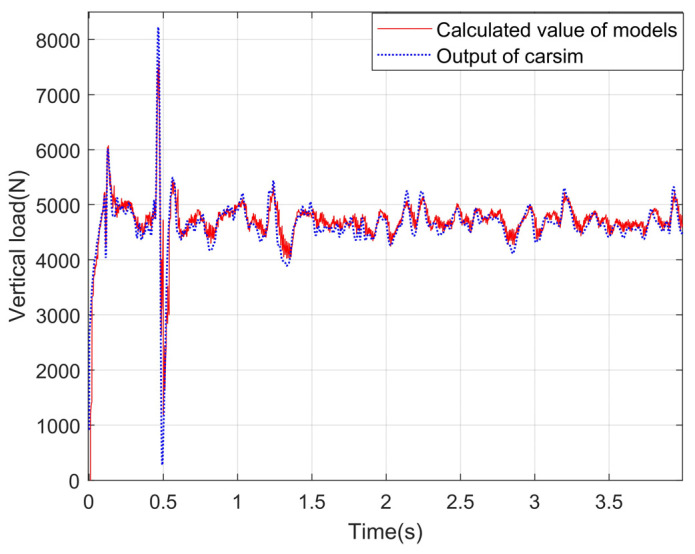
Vertical load of the right front wheel under the slope road.

**Figure 14:**
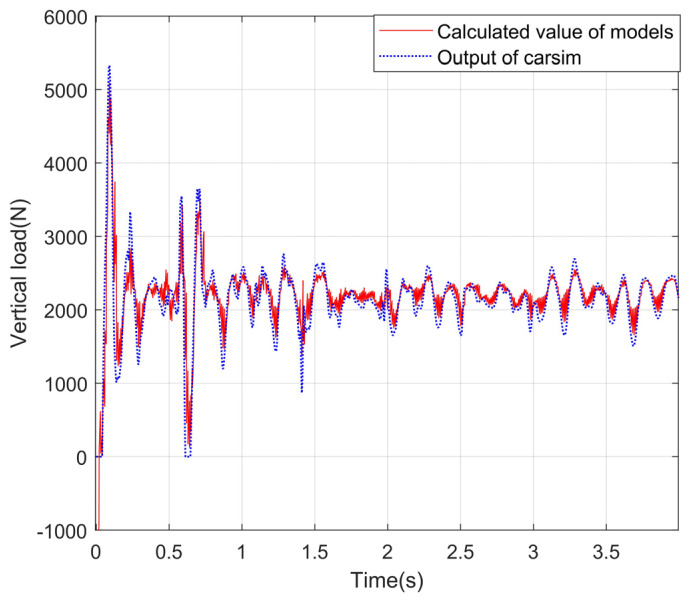
Vertical load of the left back wheel under the slope road.

**Figure 15:**
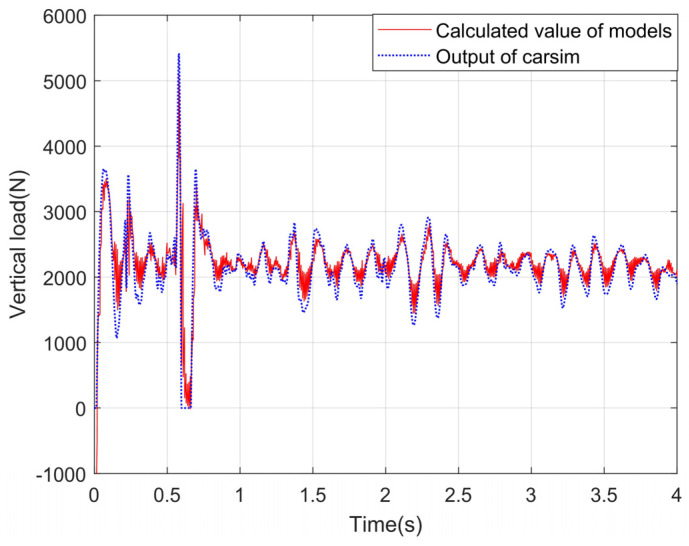
Vertical load of the right back wheel under the slope road.

A simulation environment is built in CarSim, and the vehicle response is input into the vertical load calculation module. The correctness of the model is verified by comparing the output results of the vertical load model with the output values of the vertical load in CarSim. Under the conditions of slope and uneven road, the simulation experiment is carried out, and the experimental curves are shown in Figure 12, Figure 13, Figure 14 and Figure 15.

It can be seen from the observation of the experimental curves that although some errors exist between the calculation results for the vertical load of four wheels and the corresponding CarSim outputs at the peak of the fluctuation, the changing trends are basically the same. It indicates that the model can calculate the vertical loads of the wheels more accurately on a slope and uneven road. In a word, the reason why the algorithm proposed in this paper has high precision is that the equivalent suspension model is introduced in this paper, and the load is calculated by the vertical response signals such as velocity and acceleration.

### 4.4. Experimental Results and Analysis about the Adhesion Coefficient Identification

For the two simulation scenarios described in Section 4.1.2, this section will analyze the corresponding simulation results.

#### 4.4.1. No Steering and Braking Scenario

**Figure 16:**
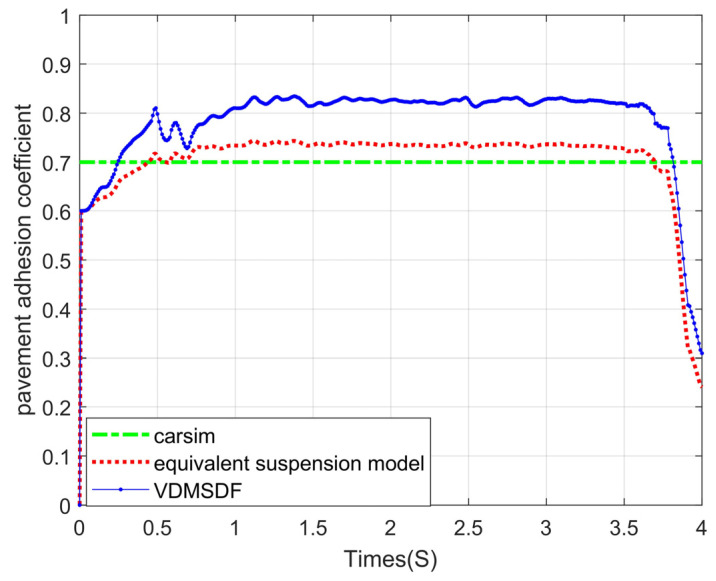
Estimated results of pavement adhesion coefficient 0.7.

When the adhesion coefficient of roads is set to 0.7, the simulation and road adhesion coefficient estimation are conducted in this part. The estimation results of the adhesion coefficients on the basis of the equivalent suspension model are compared with the conventional adhesion coefficient estimation results based on the VDMSDF. The experimental result is shown in Figure 16.

The estimated curve of the pavement adhesion coefficient based on the equivalent suspension is shown in Figure 16. It can be seen from Figure 16 that the curve converges to about 0.74 at 1 s. And then a small fluctuation is maintained until the vehicle’s braking is completed after 3 s. Based on the VDMSDF, the convergence speed of the curve in Figure 16 is slow and finally stabilizes at about 0.83. Considering that there is a large deviation between both methods, the proposed method in this paper has better estimation accuracy than the latter.

**Figure 17:**
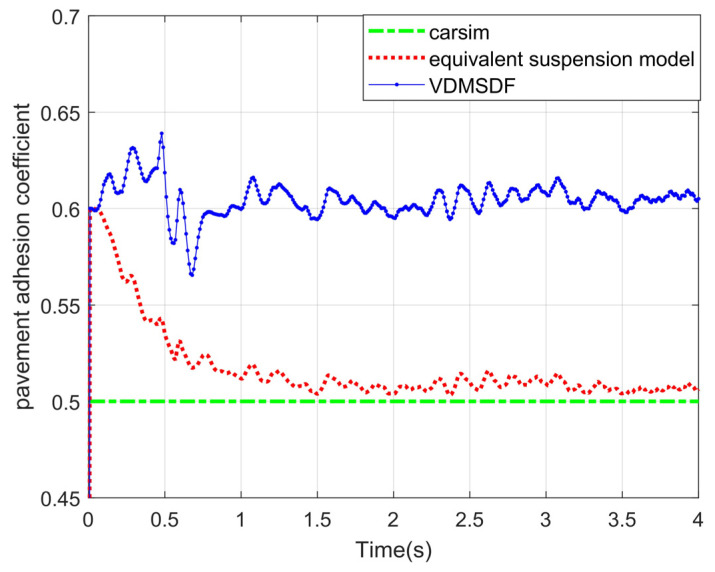
Estimated results of pavement adhesion coefficient 0.5.

Set the adhesion coefficient of roads at 0.5. The estimated curve is shown in Figure 17.

As can be seen from the experimental curve under the slope road surface in Figure 17, the adhesion coefficient estimation based on the traditional seven-degree-of-freedom vehicle dynamics model fluctuates around 0.6 and cannot converge to 0.5. The adhesion coefficient identification on the basis of the equivalent suspension converges to about 0.5 in 1 s and then has less fluctuation.

**Figure 18:**
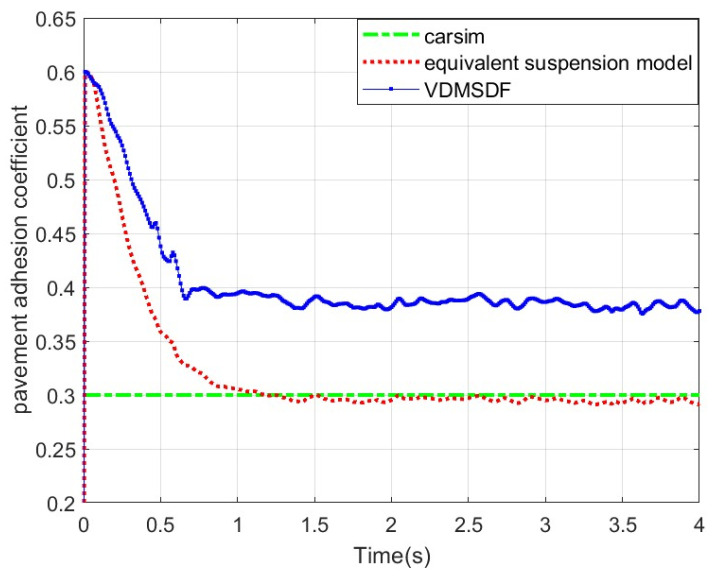
Estimated results of pavement adhesion coefficient 0.3.

Set the adhesion coefficient of roads to 0.3, and the corresponding estimated result is shown in Figure 18.

The experimental curve in Figure 18 suggests that the corresponding estimated result based on the equivalent suspension rapidly converges at 0~1 s and then remains stable at around 0.29. Based on the VDMSDF, the estimated result curve is stable around 0.39.

Generally speaking, although a certain error exists between the estimation results based on the equivalent suspension and the adhesion coefficients set by CarSim, the error is small, and the accuracy is higher than that based on the VDMSDF. The root-mean-square errors (RMSEs) of the estimation results of two algorithms are displayed in Table 4.

According to the RMSEs of the estimation results in Table 4, the equivalent suspension model-based estimation result has a smaller error under various road adhesion coefficients, and the estimation accuracy is improved by at least 3.6%. Compared with the estimation based on the VDMSDF, the estimation accuracy is higher on the non-structural road surface with its slope and uneven road surface characteristics.

#### 4.4.2. Steering and Braking Scenario

**Figure 19:**
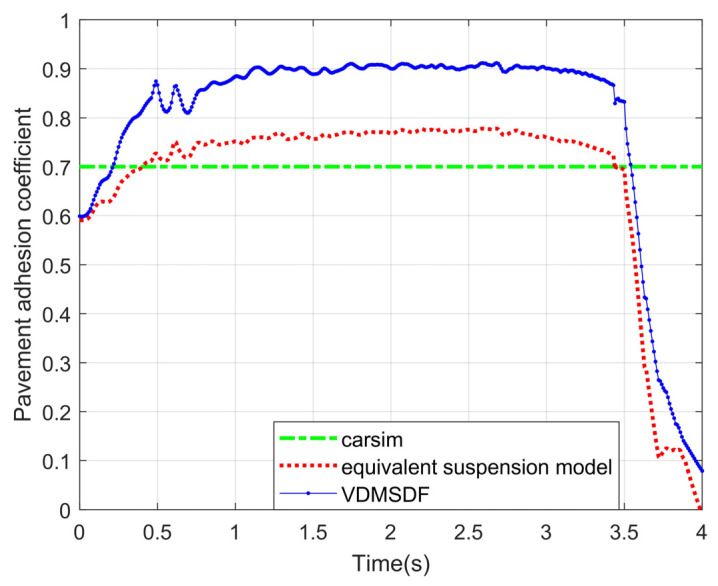
Estimated results of pavement adhesion coefficient 0.7.

The results of the road adhesion coefficient estimation based on the equivalent suspension model are also compared with those based on the seven-DOF vehicle dynamics model in Figure 19.

The experimental curve in Figure 19 indicates that the estimated result curve based on the equivalent suspension model converges to about 0.76 at 1 s and then maintains a small fluctuation until the vehicle braking is completed after 3 s. Based on the VDMSDF, the curve convergence speed is slow, and finally stabilizes at about 0.9, with a large deviation.

**Figure 20:**
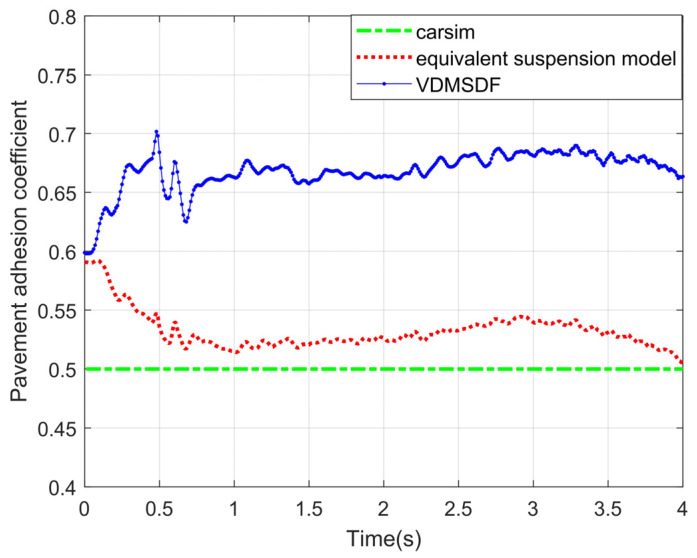
Estimated results of pavement adhesion coefficient 0.5.

The adhesion coefficient of roads is set to 0.5, and estimating results under steering braking conditions are shown in Figure 20.

The experimental curve in Figure 20 shows that the estimated result curve based on the equivalent suspension model remains stable at around 0.51. Based on VDMSDF, the estimated result curve is stable around 0.68.

**Figure 21:**
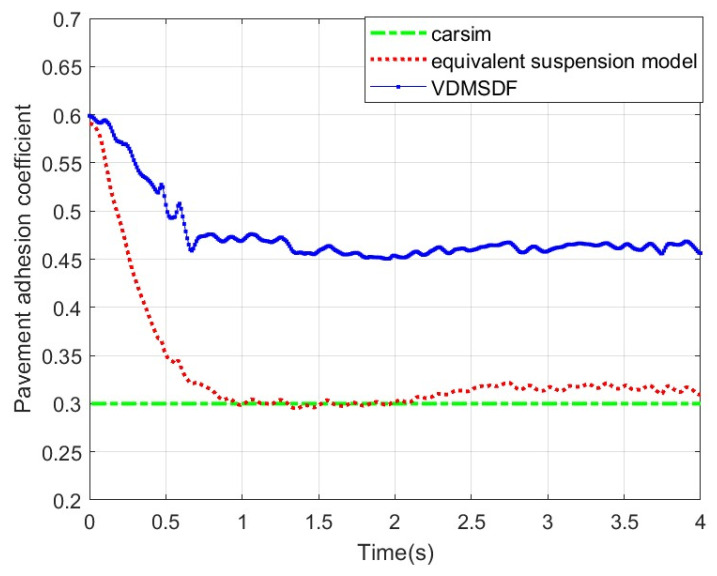
Estimated results of pavement adhesion coefficient 0.3.

The adhesion coefficient of roads is set to 0.3, and the estimated result curve under the steering braking condition is shown in Figure 21.

As shown in Figure 21, the experimental results of the equivalent suspension model and the seven-DOF vehicle dynamics model remain stable at around 0.3 and 0.45, respectively.

The root-mean-square errors (RMSEs) of the two estimation methods are calculated, and the results are shown in Table 5.

According to the RMSEs of the estimation results in Table 5, the estimation results on the basis of the equivalent suspension model have a smaller error under various road adhesion coefficients, and the estimation accuracy is improved by at least 3.7%. In a word, compared with the estimation based on the VDMSDF, the estimation accuracy is higher on the non-structural road surface under the steering braking conditions.

#### 4.4.3. Variable Adhesion Coefficient Scenario

In this scenario, the designed identification algorithm on the basis of the traditional seven-DOF vehicle dynamics model together with the proposed method in the paper is applied to identify and compare the adhesion coefficients. The experimental curves are displayed in the figure.

**Figure 22:**
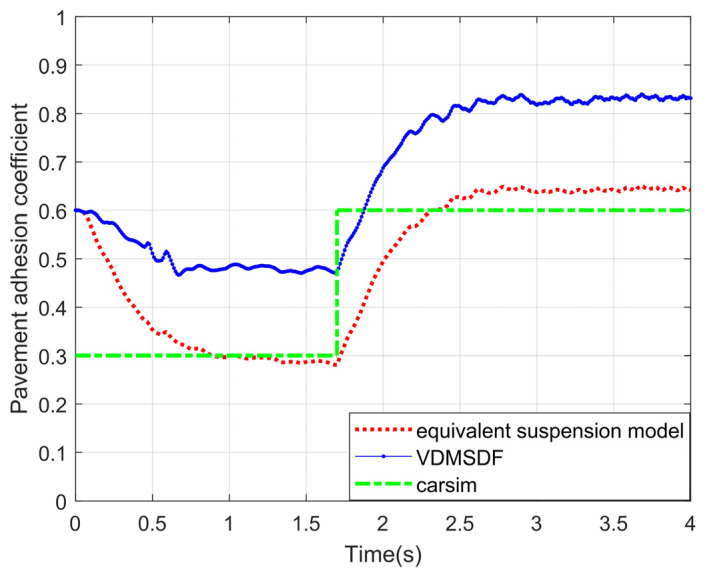
Estimated results of variable adhesion coefficient scenario.

The experimental curve in Figure 22 suggests that the proposed algorithm can identify the sudden change of adhesion coefficients with high accuracy, and the sudden change in pavement adhesion coefficients can be identified within 1.5 s.

In a word, the identification method by virtue of the equivalent suspension model has higher accuracy on the road surface with slope and road roughness characteristics, and the method has strong applicability and can be used under the straight line, the steering conditions, and the different slope conditions. The reason why the identification error of the contrast method is large is that the traditional dynamics models with seven-degrees of freedom ignore the vertical response of the vehicle; the vertical load of the wheels in the model is very different from the actual value while driving on uneven road surfaces. By introducing the equivalent suspension model, the algorithm proposed in this paper reduces the deviation of the wheel vertical load and improves identification accuracy.

## 5. Conclusions

In the study, an estimation method of adhesion coefficients on unstructured pavement by virtue of the extended Kalman filter is put forward in this paper, which can better identify the adhesion coefficient under the road with the uneven and large slope. The identification accuracy of road adhesion coefficients under unstructured pavement is improved by introducing the equivalent suspension model to optimize the calculation of vertical wheel load and modifying vehicle acceleration combined with vehicle posture data. And the multi-condition simulation experiments with CarSim prove that the proposed identification algorithm for adhesion coefficients has a higher estimation accuracy that has improved by at least 3.6%. In a word, the designed method in the paper is efficient. The identification of adhesion coefficients in the designed method is mainly applied to automatic driving scenarios. For example, the ground adhesion coefficient can be obtained and shared through the excitation response data from the driving vehicles in front, which is convenient for the path planning and stability control of subsequent vehicles.

## Figures and Tables

**Table 1:** Experimental parameter setting.

Target Speed/(km/h)	Brake Control/ MPa	Open Loop Control/Deg	Pavement Adhesion Coefficient μ	Slope
100	10	0	0.3	0.1

**Table 2:** Experimental parameter setting.

Target Speed/(km/h)	Brake Control/MPa	Open Loop Control/Deg	Pavement Adhesion Coefficient μ	Slope
100	10	0	0.7	0.1
100	10	0	0.5	0.1
100	10	0	0.3	0.1

**Table 3:** Parameter setting table of steering and braking.

Target Speed	Brake Control	Open Loop Control	Adhesion Coefficient μ
100 km/h	10 MPa	45 deg	0.7
100 km/h	10 MPa	45 deg	0.5
100 km/h	10 MPa	45 deg	0.3

**Table 4:** RMSEs of experimental estimation results.

Pavement Adhesion Coefficient μ	RMSEs of Equivalent Suspension Model	RMSEs of VDMSDF
0.7	0.05608594	0.11667209
0.5	0.03688530	0.10776193
0.3	0.069288424	0.115742819

**Table 5:** RMSEs of experimental estimation results.

Pavement Adhesion Coefficient μ	RMSEs of Equivalent Suspension Model	RMSEs of VDMSDF
0.7	0.06281493	0.17942874
0.5	0.03788459	0.16536712
0.3	0.07688491	0.18405923

## Data Availability

The data that support the findings of this study are available from the corresponding author upon reasonable request.
